# Study protocol for a prospective, multicenter, single-arm study investigating the impact of the implementation of standardized hyperkalemia management in chronic kidney disease patients

**DOI:** 10.1186/s12882-026-04801-8

**Published:** 2026-04-21

**Authors:** Yeqing Xie, Yang Li, Yimei Wang, Jing Lin, Yuxin Nie, Wuhua Jiang, Nana Song, Shuan Zhao, Ziyan Shen, Qing Li, Xiaoyan Zhang, Xiaoqiang Ding

**Affiliations:** 1 Department of Nephrology, Zhongshan Hospital, Fudan University, No. 180, Fenglin Road, Xuhui District, Shanghai, China; 2 Astrazeneca Investment (China) Co. LTD, Shanghai, China

**Keywords:** Hyperkalemia, Renin-angiotensin-aldosterone system inhibitors, Quality improvement, Disease management, Guideline adherence, Chronic kidney disease

## Abstract

**Background:**

Hyperkalemia (serum potassium > 5.0 mmol/L) is a common and clinically significant complication in patients with chronic kidney disease (CKD), particularly among those treated with renin-angiotensin-aldosterone system inhibitors (RAASi). Although guideline-directed medical therapy recommends proactive hyperkalemia management to enable continued RAASi use, real-world practice remains suboptimal. Key challenges include inadequate potassium monitoring after RAASi initiation or dose escalation, unnecessary RAASi dose reduction or discontinuation following hyperkalemia episodes, and poor long-term treatment adherence. Moreover, evidence regarding the cardio-renal benefits of RAASi optimization supported by structured hyperkalemia management in Chinese CKD populations is limited. This study aims to bridge the gap between Kidney Disease: Improving Global Outcomes (KDIGO) and Chinese CKD guideline recommendations and routine clinical practice through a nationwide quality improvement program (QIP).

**Methods:**

This prospective, multicenter, single-arm study will enroll approximately 1,000 adult Chinese patients with non-dialysis CKD and hyperkalemia across 50 sites. The QIP includes comprehensive and reinforcement training for healthcare professionals (HCPs) based on the KDIGO 2024 guidelines, supported by regular quality audits and feedback. Patient-directed interventions comprise structured education delivered by HCPs and self-learning modules. Participants will receive standardized hyperkalemia management over 96 weeks, including protocolized serum potassium monitoring (every 3 months or monthly for high-risk patients), RAASi dose optimization, and long-term potassium control. The primary endpoint is the proportion of patients achieving RAASi optimization with normokalemia (3.5-5.0 mmol/L) at 48 weeks. Secondary endpoints include sustained potassium control, RAASi optimization over time, medication adherence, hyperkalemia recurrence and severity, cardiorenal outcomes, and safety endpoints.

**Discussion:**

As the first nationwide QIP focusing on hyperkalemia management in CKD patients in China, this study is expected to provide real-world evidence on the effectiveness of standardized hyperkalemia care in facilitating sustained RAASi use. By aligning clinical practice with guideline recommendations, the program has the potential to improve long-term cardio-renal outcomes and inform future CKD care strategies.

**Trial registration:**

ClinicalTrials.gov, NCT06884267; registered March 13, 2025.

**Supplementary Information:**

The online version contains supplementary material available at 10.1186/s12882-026-04801-8.

## Background

Hyperkalemia, defined as a serum potassium level > 5.0 mmol/L, is one of the most common and clinically significant electrolyte abnormalities encountered in clinical practice [1, 2]. The kidneys play a central role in maintaining potassium homeostasis through filtration, reabsorption, and secretion; consequently, disturbances in potassium balance are particularly prevalent in patients with chronic kidney disease (CKD) owing to reduced glomerular filtration rate and tubular dysfunction [3]. In addition, treatment with renin-angiotensin-aldosterone system inhibitors (RAASi), a cornerstone therapy in CKD, further increases the risk of hyperkalemia by inhibiting aldosterone-mediated renal potassium excretion [4]. The reported prevalence of hyperkalemia (> 5.0 mmol/L) among patients with CKD ranges from 8% to 38% [5]. In China, a large outpatient survey demonstrated that while 3.86% of the general outpatient population experienced hyperkalemia, the prevalence increased markedly to 22.89% among individuals with CKD [6]. Importantly, even mild-to-moderate elevations in serum potassium have been consistently associated with increased short- and long-term mortality, higher rates of cardiac arrhythmias, and adverse hospital outcomes in patients with CKD or cardiovascular disease [7, 8]. These risks are further amplified in patients with severe hyperkalemia or comorbid conditions such as advanced CKD or acute kidney injury, underscoring the need for vigilant potassium monitoring and proactive management strategies [8–10].

In patients with CKD, this risk of hyperkalemia frequently leads to conservative prescribing practices, including suboptimal dosing or premature discontinuation of RAASi therapy in routine clinical care [11–13]. However, extensive evidence demonstrates that RAASi therapy confers substantial clinical benefit, including reductions in blood pressure and proteinuria, slowing of estimated glomerular filtration rate (eGFR) decline, and significant decreases in the risks of kidney failure, cardiovascular morbidity, and all-cause mortality among patients with CKD [14]. Importantly, observational analyses have shown that RAASi discontinuation following hyperkalemia is associated with markedly increased risks of mortality, cardiovascular events, and progression to end-stage kidney disease (ESKD), underscoring the cardioprotective and renoprotective consequences of sustained RAASi exposure [15]. In recognition of these benefits, contemporary clinical guidelines emphasize that hyperkalemia should be actively managed to enable continuation of RAASi therapy whenever possible. The Kidney Disease: Improving Global Outcomes (KDIGO) 2024 guideline recommends a stepwise approach to hyperkalemia management in CKD, prioritizing correction of reversible factors, optimization of using potassium-lowering agents before considering dose reduction or discontinuation of RAASi or mineralocorticoid receptor antagonists as a last resort [16, 17].

Despite clear and consistent guideline recommendations, a substantial gap persists between evidence-based guidance and real-world hyperkalemia management in patients with CKD. In routine clinical practice, serum potassium monitoring remains insufficient, and adherence to long-term hyperkalemia management strategies is often suboptimal, limiting the ability to maintain guideline-recommended RAASi therapy [11–13]. Large-scale observational studies indicate that more than half of eligible CKD patients receive lower-than-recommended RAASi doses, while approximately 14–16% discontinue RAASi therapy altogether [11]. From the patient perspective, inadequate disease awareness, poor adherence to potassium monitoring schedules, and challenges in sustaining long-term treatment regimens represent key barriers. From the clinician perspective, concerns regarding hyperkalemia risk, variability in treatment approaches, and the absence of a unified, standardized management pathway frequently result in conservative RAASi prescribing, including dose reduction or discontinuation, even when alternative potassium-lowering strategies are available [11–13]. Moreover, although guideline-directed medical therapy emphasizes proactive hyperkalemia management to preserve RAASi exposure, robust real-world evidence demonstrating the long-term cardiovascular and renal benefits of sustained, guideline-based hyperkalemia care remains limited, particularly in the Chinese CKD population.

Therefore, this study was designed to evaluate the impact of implementing a standardized, guideline-directed hyperkalemia management strategy through a structured quality improvement program (QIP). By targeting both healthcare professionals (HCPs) and patients, the study aims to bridge the gap between guideline recommendations and routine clinical practice, improve serum potassium monitoring and control, facilitate RAASi optimization, and generate real-world evidence on the long-term cardiorenal benefits of consistent hyperkalemia management in patients with CKD.

## Methods/design

### Study design

**Fig. 1:**
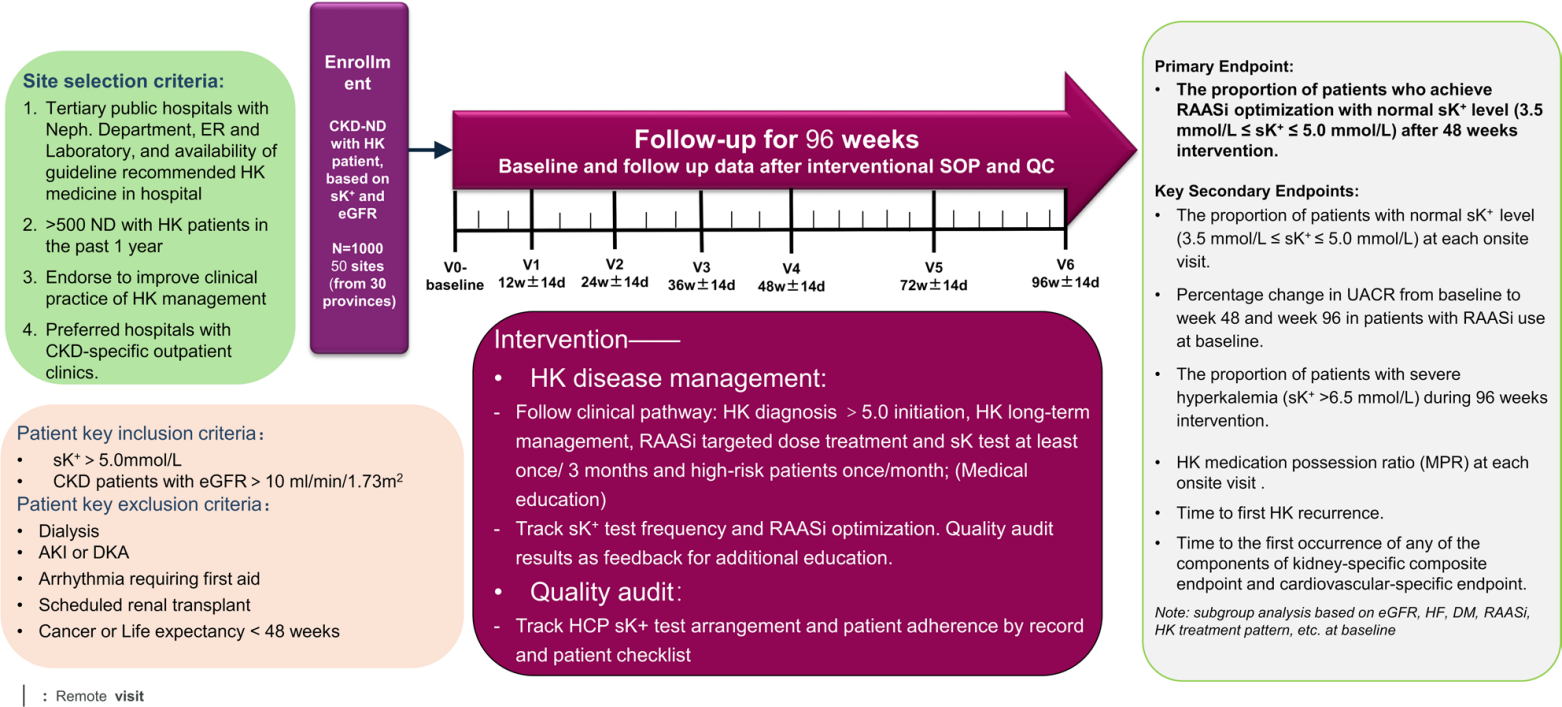
Study design

This multicenter, prospective, single-arm study (ClinicalTrials.gov identifier: NCT06884267) is designed to evaluate the impact of a QIP targeting both HCPs and patients, based on the KDIGO 2024 recommendations, in individuals with CKD and hyperkalemia. The QIP aims to determine whether implementation of a structured, guideline-based intervention can improve care processes and clinical outcomes in routine practice. The overall study design is illustrated in Fig. 1.

## Study sites and participants

### Site selection

Approximately 50 research centers across China will be selected based on the following criteria: (1) tertiary public hospitals with an established Department of Nephrology, emergency services, and laboratory facilities, and with access to guideline-recommended treatments for hyperkalemia; (2) management of more than 500 non-dialysis patients with CKD and hyperkalemia within the preceding year; (3) demonstrated institutional commitment to improving hyperkalemia management; and (4) preferential inclusion of hospitals with CKD-specific outpatient clinics.

### Participants

Eligible participants must be ≥ 18 years of age at the time of providing written informed consent and be capable of complying with the protocol and informed consent form requirements. Participants must have a diagnosis of CKD with an eGFR > 10 mL/min/1.73 m², calculated using the Chronic Kidney Disease Epidemiology Collaboration (CKD-EPI) 2021 creatinine equation. In addition, a serum potassium level > 5.0 mmol/L documented within 48 h prior to enrollment is required. Exclusion criteria include receipt of dialysis therapy, pseudo-hyperkalemia, acute kidney injury, diabetic ketoacidosis, or cardiac arrhythmias requiring immediate medical intervention. Patients with a history of renal transplantation or those scheduled to undergo renal transplantation will also be excluded. A complete list of inclusion and exclusion criteria is provided in the Supplementary Material.

### Endpoints

Both the KDIGO 2024 and Chinese CKD guidelines recommend the use of RAASi at the maximum tolerated dose to achieve optimal therapeutic benefits, including cardiorenal protection [17–19]. However, in routine clinical practice, RAASi therapy is frequently down-titrated or discontinued in response to hyperkalemia, often in deviation from guideline recommendations. Accordingly, the primary endpoint of this study is designed to capture not only effective potassium control in patients with CKD and hyperkalemia, but also successful RAASi optimization. The primary endpoint is defined as the proportion of patients achieving RAASi optimization while maintaining normokalemia (serum potassium 3.5-5.0 mmol/L) at 48 weeks following implementation of the intervention.

The secondary endpoints include: (1) the proportion of patients with normokalemia (serum potassium 3.5-5.0 mmol/L) at each onsite visit; (2) the proportion of patients achieving RAASi optimization with normokalemia at weeks 12 and 24; (3) the percentage change in urinary albumin-to-creatinine ratio (UACR) from baseline to weeks 48 and 96 among patients receiving RAASi at baseline; (4) the proportion of patients experiencing severe hyperkalemia (serum potassium > 6.5 mmol/L) during the 96-week intervention period; (5) medication possession ratio (MPR) for hyperkalemia therapies, defined as the number of days hyperkalemia medication was used between two onsite visits divided by the total number of days between those visits, assessed at each onsite visit; (6) time to first hyperkalemia recurrence; (7) hospital-level hyperkalemia management status at baseline and at weeks 48 and 96; (8) time to first occurrence of any component of the kidney-specific composite endpoint, defined as sustained eGFR decline ≥ 50%, ESRD, or renal death; (9) eGFR slope over the 96-week follow-up period; (10) time to first occurrence of any component of the heart-specific composite endpoint, including rehospitalization for heart failure (rhHF), worsening heart failure, emergency room visits, or cardiovascular death; (11) time to first occurrence of arrhythmia; (12) frequency of serum potassium testing and number of hyperkalemia episodes during the first 48 weeks of intervention (onsite and remote visits), with hyperkalemia defined as serum potassium > 5.0 mmol/L and multiple measurements within 48 h counted as a single episode; (13) the proportion of patients requiring emergency department visits, emergency dialysis, or hospitalization during the 48-week intervention period; (14) safety and clinical assessments, including adverse events (AEs), serious adverse events (SAEs), adverse drug reactions (ADRs); (15) vital signs; (16) physical examination findings; (17) clinical laboratory parameters; and (18) electrocardiographic assessments.

### Timeline of the study

The schedules of study activities for patients and participating sites are summarized in Tables 1 and 2, respectively. Patient enrollment will commence only after completion of comprehensive guideline-directed medical therapy training for HCPs at both the study-wide and individual site levels. The screening period will extend from 14 days to 1 day prior to enrollment. Following enrollment, patients will receive individualized treatment plans and hyperkalemia management strategies determined by their HCPs, including disease education, self-management materials, prescriptions for RAASi, serum potassium monitoring, and potassium-lowering therapies as appropriate. The maximum duration of follow-up will be 96 weeks. After the baseline visit and initial data collection, each participant will attend six scheduled onsite visits at weeks 12, 24, 36, 48, 72, and 96 post-enrollment. Patients who discontinue participation prematurely will be invited to complete an early withdrawal visit. During intervals without scheduled onsite visits, participants will undergo remote follow-up every 4 weeks. Patients will be considered to have completed the study upon completion of the 96-week follow-up, death, or study termination. The study end date is defined as the date of the final visit of the last enrolled patient.

**Table 1:** Schedule of activities for patients

Procedure	Screening period	Management period/follow up^a^							Early Withdrawal + 14 day^c^
Visit ^b^	Day − 14 ~ Day-1	Day 0 Baseline *	Week 12 ± 14 days	Week 24 ± 14 days	Week 36 ± 14 day	Week 48 ± 14 days	Week 72 ± 28 days	Week 96 ± 28 days	
	/	V0	V1	V2	V3	V4	V5	V6	
Informed consent	X								
Inclusion and exclusion criteria	X	X							
Demographics ^d^	X								
Medical history ^e^	X								
Physical examination ^f^	X		X	X	X	X	X	X	
Height, weight	X	X	X (weight only, if available as per clinical practice)						
Laboratory tests (Hematology, Clinical Chemistry, Urinalysis)	X^g^		X	X	X	X	X^h^	X^h^	X^i^
12-lead ECG ^j^	X	X	X	X	X	X	X	X	X
Vital signs ^k^	X	X	X	X	X	X	X	X	
Patient’s daily checklist release and/or retrieve		X	X	X	X	X	X	X	X
RAASi treatment pattern ^l^		X	X	X	X	X	X	X	X
Hyperkalemia treatment pattern ^m^		X	X	X	X	X	X	X	X
Concomitant medication ^n^	X	X	X	X	X	X	X	X	X
Clinical progression/medical events collection			X						X
Administration of study intervention		X	X •Patient education at each onsite visit •Patient self-learning						
AE/SAE^p^	X								X

HCP, health care professional; BP, blood pressure; eGFR, estimated glomerular filtration rate; ECG, electrocardiogram; RAASi, renin-angiotensin-aldosterone system inhibitor; UACR, urinary albumin/creatinine ratio; ESRD, end-stage renal disease; rhHF, rehospitalization for heart failure; HF, heart failure; ER, emergency room; CV-death, cardiovascular death; AE, adverse event; SAE, serious adverse event; ICF, informed consent form; MAH, marketing authorization holder

a. Day 0 refers to the day on which the patient is enrolled

b. Remote visits: Telephone calls will be conducted every 4 weeks (± 7 days) following enrollment if no onsite visit occurs, to review patient self-management and assess adherence. Patients will also be reminded to follow their HCP’s advice regarding serum K⁺ testing, medication adherence, dietary restrictions, etc

c. Early withdrawal visits will be conducted for patients who discontinue early, preferably within two weeks of withdrawal confirmation. These visits may be onsite or remote

d. Demographic data include gender, age, race, and ethnicity

e. Medical history includes comorbidities and relevant conditions requiring medication within 1 year prior to enrollment

f. Physical examination: A complete physical examination will be performed at V0 (Day 0). A brief physical examination will be conducted during follow-up visits. A complete physical examination includes assessment of general appearance; respiratory, cardiovascular, abdominal, skin, head and neck (including ears, eyes, nose, and throat); lymph nodes; thyroid; musculoskeletal (including spine and extremities); and neurological systems. A brief physical examination includes assessment of at least the neurological system, skin, lungs, cardiovascular system, abdomen (liver and spleen), and lymph nodes

g. The serum K⁺ test must be completed within 48 h prior to enrollment. Other laboratory test results within 14 days before baseline may be accepted

h. At weeks 72 and 96, only hematology, urinalysis, renal function (eGFR), blood electrolytes (Na⁺/K⁺/Cl⁻), and UACR will be assessed. Additional laboratory tests may be collected if available per routine clinical practice

i. At early withdrawal visits, only renal function (eGFR) and serum K⁺ will be tested. Additional laboratory assessments may be performed if available per clinical practice

j. Additional ECGs may be performed if available as per clinical practice. ECG results obtained within 14 days prior to baseline may be used as screening data

k. Vital signs include body temperature, systolic and diastolic blood pressure, pulse rate, and respiratory rate

l. RAASi treatment patterns include whether therapy was initiated and, if so, detailed data (drug/treatment name, usage and dosage, frequency, start and end dates, any changes or interruptions, and reasons for discontinuation, if applicable)

m. Hyperkalemia treatment: Pharmacological treatment includes whether therapy was administered and, if so, treatment name, usage and dosage, frequency, start and end dates, and any changes or interruptions with reasons (if applicable). Non-pharmacological treatment includes treatment name, start and end dates, and reason for change or interruption (if applicable)

n. Concomitant medications will be collected throughout the study. Blood pressure and RAASi treatment in the 3 months prior to enrollment will also be recorded. For patients continuing RAASi treatment during screening, the date of the last dose prior to enrollment should be documented

o. Clinical progression/medical events include: Kidney-specific composite events: sustained eGFR decline ≥ 50%, ESRD, or renal-related death. Cardiovascular-specific composite events: rhHF, worsening HF, emergency room visit, or cardiovascular death. Other events: arrhythmia, hospitalization, emergency dialysis, emergency department visit, and death due to other causes

p. Adverse events (AEs): All AEs and SAEs related to potassium binders, as well as special situations (e.g., medication error, drug abuse, misuse, and pregnancy) involving LOKELMA^®^, will be actively collected. The collection period spans from the time of ICF signing (for patients using a potassium binder prior to enrollment) or the date of treatment initiation (for those starting after enrollment) until 24 h post-discontinuation. AEs involving other products for which AstraZeneca is not the marketing authorization holder (MAH) will be reported spontaneously to the respective MAHs

* Demographic and medical history data collected during the screening period may be used as baseline data

**Table 2:** Schedule of activities for sites

Procedure	Before first patient is enrolled in the study	Before first patient is enrolled in the site	FPI + 4 weeks	FPI + 12 weeks	FPI + 16 weeks	FPI + 24 weeks	FPI + 28 weeks	FPI + 36 weeks	FPI + 40 weeks	FPI + 48 weeks	FPI + 96 weeks
GDMT comprehensive training	X LPI to site investigator	X Site investigator to HCPs									
Mandatory intensive training ^a^			X		X		X		X		
Added intensive training				If off target, ≤ 4 times/site during 0–48 weeks after FPI, totally ≤ 6 times/site, at the individual site level							
Hyperkalemia management status of sites (questionnaire)	X^b^									X^b^	X^b^
Quality audit at the HCP level				X FPI + 3 months		X FPI + 6 months		X FPI + 9 months	X FPI + 12, 15, 18 months		

GDMT, guideline determined medical therapy; PI, principal investigator; HCP, health care professional

a. FPI refers to “first patient in” (unless otherwise specified, at the site level). Mandatory intensive training at the study level (delivered by the leading PI to the site investigator) was conducted at weeks 4, 16, 28, and 40 following first patient enrollment in the study. Mandatory intensive training at the site level (delivered by the site investigator to the site’s HCPs) was conducted at weeks 4, 16, 28, and 40 following first patient enrollment at the individual site

b. A site-level questionnaire assessing hyperkalemia management status was required at baseline and at weeks 48 and 96 after first patient enrollment at each individual site

### Intervention

**Fig. 2:**
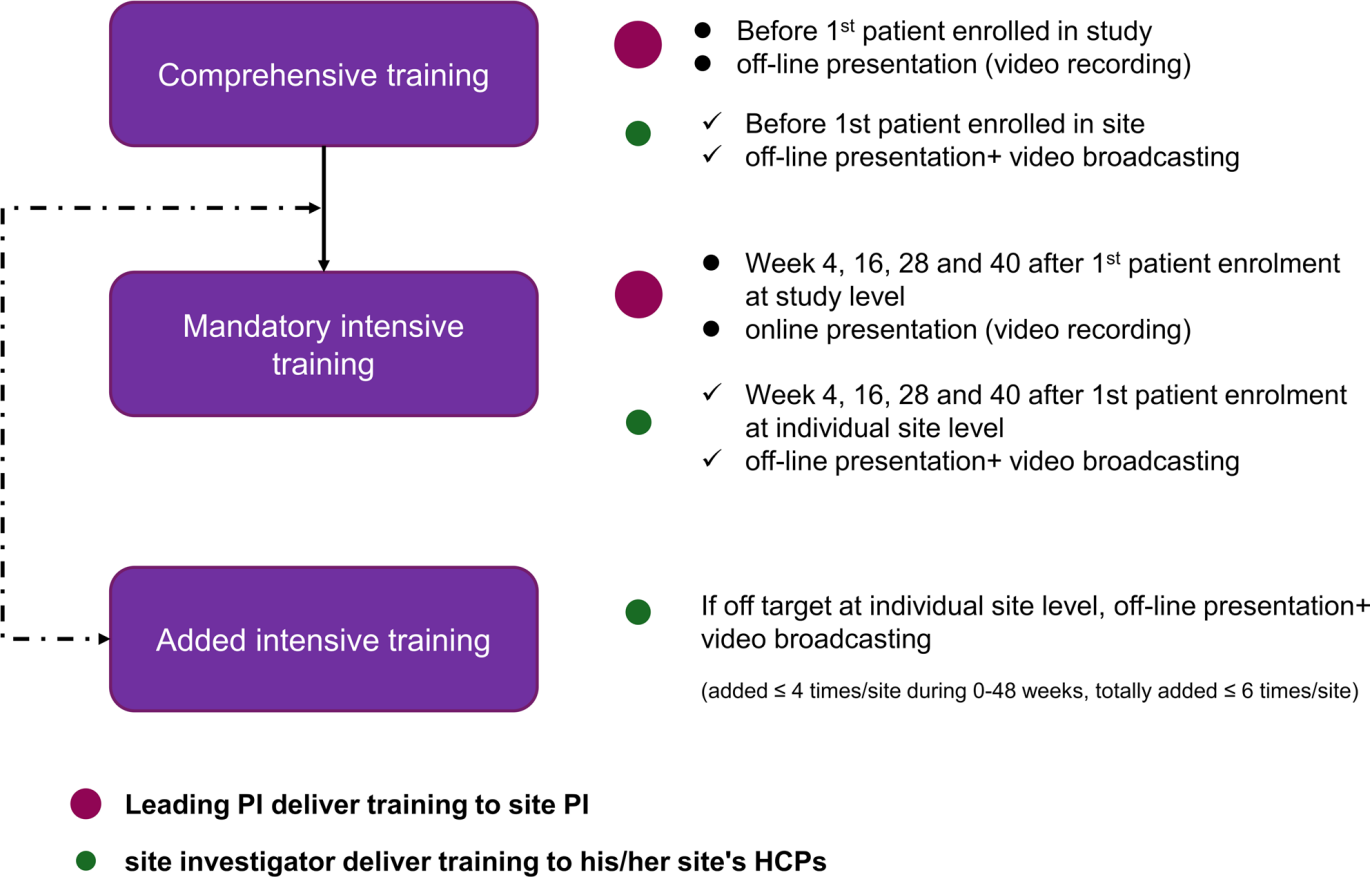
Guideline-directed management algorithm for hyperkalemia in chronic kidney disease

The study will implement a QIP targeting both HCPs and patients, incorporating standardized hyperkalemia management and ongoing quality audits. The methods and processes of the intervention are outlined in Table 3. Core components of hyperkalemia management include a standardized clinical pathway based on guideline-directed medical therapy, encompassing the use of serum potassium > 5.0 mmol/L as the diagnostic criterion, long-term hyperkalemia management strategies, targeted dosing of RAASi, and protocolized serum potassium monitoring at least every 3 months, or monthly in patients at high risk of hyperkalemia, including those with CKD combined with diabetic kidney disease, CKD combined with heart failure, or CKD undergoing initiation or uptitration of RAASi therapy. Both serum potassium testing frequency and RAASi optimization will be systematically tracked throughout the study. The standardized hyperkalemia management protocol for patients with CKD will be implemented as illustrated in Fig. 2, with the tiered management approach detailed in Table 4. The guideline-directed medical therapy framework is informed by the KDIGO 2024 Clinical Practice Guideline for the Evaluation and Management of Chronic Kidney Disease [17], the Expert consensus on the management of serum potassium in chronic kidney disease patients in China (2020) [18], and the Guidelines for the early evaluation and management of chronic kidney disease in China (2023) [19].

**Table 3:** Method and process of intervention

	HCP level	Patient level
Hyperkalemia disease management	Key contents: • Follow clinical pathway: hyperkalemia diagnosis >5.0 mmol/L, hyperkalemia long-term management^#^, RAASi targeted dose treatment and serum K^+^ test at least once per 3 months and high-risk cases^*^ once per month • Track serum K^+^ test frequency and RAASi optimization. Quality audit results as feedback for additional education. Frequency: • 1 comprehensive training • 4 mandatory intensive trainings (every 12w with the 1st at week 4) • ≤6 additional intensive trainings if quality audit is off-target	Key contents: • Disease introduction, poor outcomes and the importance of serum K^+^ monitoring Frequency: • 6 sessions of disease and medication adherence education from HCPs during onsite visits; self-learning
Quality Audit	Evaluate whether HCPs’ perception and action of standardized hyperkalemia management achieve the target. • Questionnaires to survey HCPs’ standardized hyperkalemia management perception • Track serum K^+^ test arrangement to check if arranged	Review patient checklist and self-management to check patient’s adherence and HCP’s clinical behavior

HCP, health care professional

^#^ Hyperkalemia long-term management: Hyperkalemia pharmacological treatment and hyperkalemia non-pharmacological treatment

^*^ High risk hyperkalemia patients: CKD with diabetic kidney disease; CKD with heart failure; CKD on RAASi

**Table 4:** Actions to manage hyperkalemia in chronic kidney disease

1st line: Correctable factors	• Review non-RAASi medications (e.g., NSAIDs, trimethoprim) • Assess dietary potassium intake (dietary referral) and consider appropriate moderation of dietary potassium intake
2nd line: Medications	Consider: • Appropriate use of diuretics • Optimize serum bicarbonate levels • Licensed potassium exchange agents
3rd line: Last resort	• Reduce dose or discontinue RASi/MRA (Discontinuation is associated with increased cardiovascular events. Review and restart RASi or MRA later if the patient’s condition allows.)

RAASi, renin-angiotensin-aldosterone system inhibitor; NASID, nonsteroidal anti-inflammatory drug; RASi, renin-angiotensin system inhibitor; MRA, mineralocorticoid receptor antagonist

Quality audits will be conducted at both the HCP and patient levels. At the HCP level, audits will be performed quarterly beginning after enrollment of the first patient at each site. Relevant audit data will be regularly extracted and provided as structured feedback to HCPs and site investigators, who will review the findings to promote continuous self-assessment and improvement in care processes. HCPs’ perceptions and implementation of standardized hyperkalemia management will be evaluated quarterly to assess alignment with predefined performance targets. HCP-level quality audits will assess: (1) provision of disease education and self-management materials to patients; (2) prescription practices related to RAASi, serum potassium testing, and potassium-lowering therapies; (3) scheduling of follow-up visits in accordance with the standardized clinical pathway and guideline-directed medical therapy; and (4) administration of questionnaires evaluating HCPs’ perceptions and practices regarding hyperkalemia management. Patient adherence and corresponding HCP clinical behavior will be evaluated through review of patient daily checklists every 12 weeks and self-management reports every 4 weeks. Patient-level quality audits will assess: (1) whether potassium-lowering therapy is prescribed by the designated HCP or self-selected by the patient; (2) whether RAASi therapy is prescribed by the designated HCP or self-selected by the patient; (3) whether serum potassium testing is arranged by the HCP or initiated by the patient; and (4) whether education is received through direct instruction from the designated HCP or via self-learning materials.

**Fig. 3:**
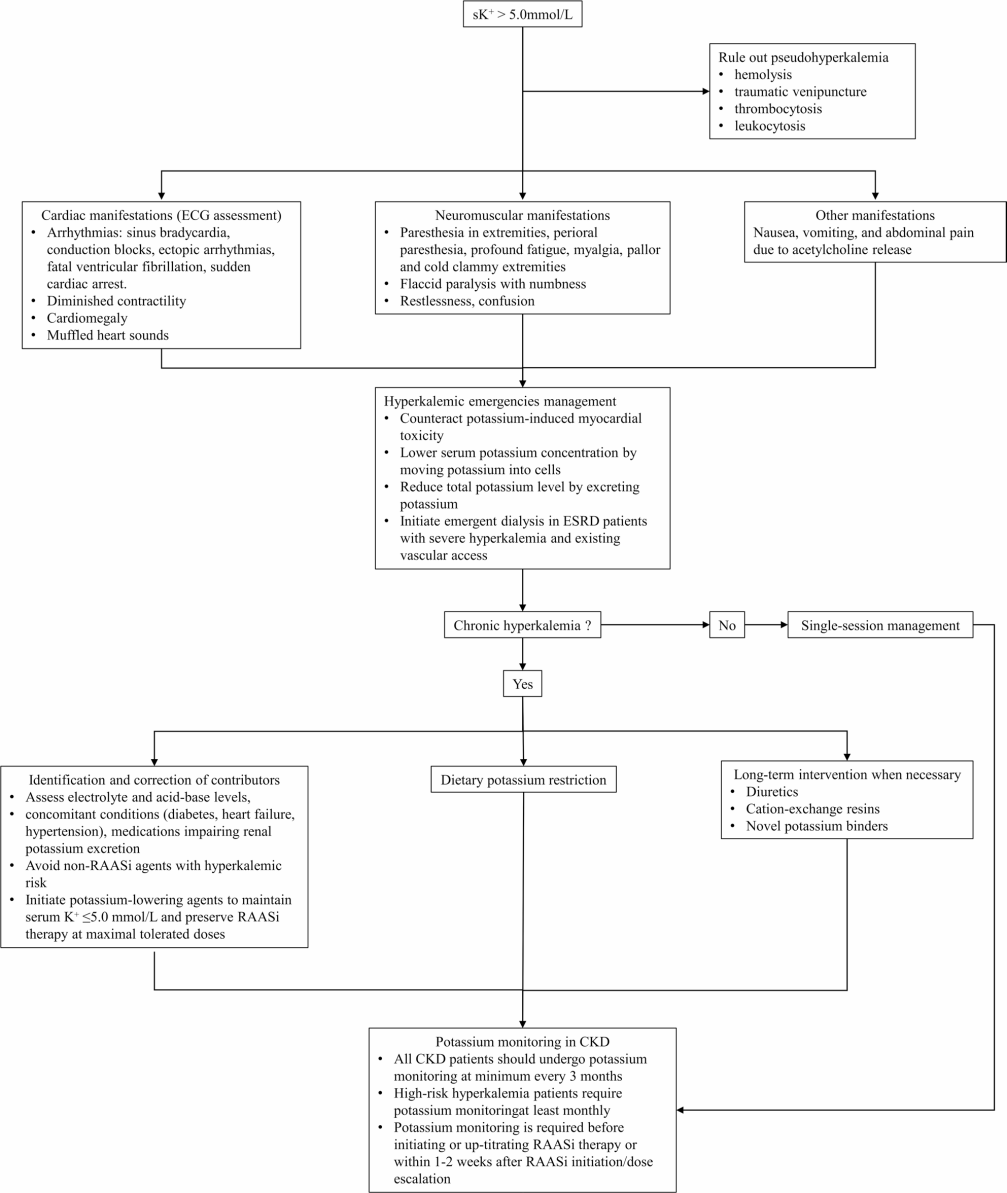
Overview of the GDMT training program. PI, principal investigator; HCP, health care professional; GDMT, guideline determined medical therapy

Audit results will be used to guide additional targeted medical education, with training serving as a key mechanism to improve hyperkalemia management at both the HCP and patient levels. HCP education focuses on guideline-directed medical therapy and includes instruction on the study design, protocol implementation, core patient management materials (Table S1), and standardized clinical pathway resources (Figure S1). HCP training comprises one comprehensive training session conducted prior to enrollment of the first patient, followed by four mandatory intensive training sessions at weeks 4, 16, 28, and 40, delivered at both the overall study and individual site levels. If quality audit targets are not met, additional site-level intensive training sessions will be implemented, with a maximum of one session per site per quarter during the first 48 weeks after enrollment of the first patient and a total cap of six additional sessions per site. These sessions will be delivered by the site investigator to local HCPs and scheduled between the mandatory intensive training sessions. Details regarding training timelines, formats, and responsible personnel are provided in Fig. 3. Patient education consists of structured disease education and medication adherence instruction delivered by HCPs, supplemented by self-learning materials. At each onsite visit from weeks 12 to 96 (weeks 12, 24, 36, 48, 72, and 96), participating HCPs will reinforce education on disease knowledge, potential adverse outcomes, and the importance of regular serum potassium monitoring. Patients will be supported in recording hyperkalemia self-management information through a dedicated patient-facing mobile application that includes educational push notifications and simplified self-management recording modules; in addition, telephone reminders will be conducted every 4 weeks during remote visits to reinforce healthcare professional guidance on serum potassium testing, medication adherence, and dietary management.

### Assessment and data collection

Demographic data will include age, sex, race, and ethnicity. Medical history will capture comorbid conditions and clinically relevant diagnoses requiring pharmacological treatment within 1 year prior to enrollment. Laboratory assessments will include serum potassium, UACR, and eGFR, with UACR calculated from measured urinary albumin and creatinine concentrations.

Patterns of RAASi use will be documented, including whether therapy was initiated or continued and, where applicable, the specific drug name, dosage, frequency, start and end dates, and any dose modifications or interruptions, together with the underlying reasons. Pharmacological treatments for hyperkalemia will be recorded in a similar manner, including treatment details and reasons for discontinuation or interruption. Non-pharmacological hyperkalemia management strategies will also be documented, including the intervention type, start and end dates, and reasons for any changes or discontinuation. Clinical progression and medical events will include kidney-specific composite outcomes (sustained eGFR decline ≥ 50%, ESRD, or renal-related death), cardiovascular-specific composite outcomes (rhHF, heart failure-related emergency visits, or cardiovascular death), as well as arrhythmias, hospitalizations, emergency dialysis visits, and death from other causes. In addition, the hyperkalemia management status of each participating site will be assessed using a site-level questionnaire administered at baseline and at weeks 48 and 96 following enrollment of the first patient (Table 2).

Safety will be monitored throughout the study period. A comprehensive baseline assessment will be conducted for each participant, including demographic characteristics, medical history, electrocardiograms, laboratory parameters, vital signs, height, weight, and physical examination findings. During the study, vital signs, electrocardiograms, and laboratory assessments will be performed according to the schedule specified in Table 1. AEs, SAEs, and concomitant medication use will be continuously monitored and systematically recorded. Each reported SAE will be individually reviewed to assess its potential impact on participant safety and the overall conduct of the study. Cumulative AEs will be tracked throughout the study, and all AEs will be followed until resolution, stabilization, or completion of the end-of-study visit.

### Sample size estimation

The study is expected to enroll approximately 1,000 patients, based on the estimated eligible patient population across China and the participation of approximately 50 research centers over an anticipated enrollment period of 10 months. As this study is descriptive in nature, the sample size was determined to ensure adequate precision of estimates rather than formal hypothesis testing. Assuming that 45% of patients will achieve RAASi optimization while maintaining normokalemia (serum potassium 3.5-5.0 mmol/L) at 48 weeks [20], and allowing for an anticipated 20% dropout rate, a total sample size of 1,000 patients is expected to provide a precision of 3.4% for the primary endpoint, calculated using the normal approximation method.

### Statistical plans

All statistical analyses will be performed using SAS software (version 9.4 or later). Analyses will be primarily descriptive in nature and will be conducted using non-missing data only.

Except for safety analyses, all efficacy and exploratory analyses will be performed in the full analysis set (FAS), which includes all patients who meet the inclusion and exclusion criteria, unless otherwise specified. Continuous variables will be summarized using the number of observations, mean, standard deviation, median, first and third quartiles, minimum, and maximum. Categorical variables will be summarized as frequencies and percentages. 95% confidence intervals (CIs) will be reported where appropriate. For time-to-event endpoints, analyses will include the number and proportion of patients experiencing each event, along with the corresponding event rates. Kaplan-Meier methods will be used to estimate and graphically present cumulative incidence for both composite and individual endpoints. Changes in eGFR over time will be evaluated using a mixed-effects model for repeated measures (MMRM) to estimate the eGFR slope over the study period.

Safety analyses will be conducted in the safety analysis set (SAS), which includes all patients who have received at least one potassium-binding therapy. The number and proportion of patients experiencing at least one AE, SAE, or ADR will be summarized. Vital signs at each scheduled assessment, as well as findings from physical examinations, electrocardiograms, and laboratory evaluations, will be summarized using descriptive statistics.

A total of four interim analyses are planned, including one primary analysis. The primary analysis is scheduled to occur 48 weeks after enrollment of the final patient.

In addition, predefined subgroup analyses will be performed for the primary and selected secondary endpoints. Subgroups will be defined based on demographic characteristics, baseline disease features (including baseline eGFR to reflect CKD stage), geographic region, and other clinically relevant factors such as history of heart failure, diabetes mellitus, RAASi use, and hyperkalemia treatment patterns.

### Ethical considerations

The study will be conducted in accordance with internationally recognized ethical principles, including the Declaration of Helsinki (revised at the 64th World Medical Association General Assembly, Fortaleza, Brazil, October 2013) and the Council for International Organizations of Medical Sciences (CIOMS) International Ethical Guidelines. The International Council for Harmonisation Good Clinical Practice guidelines, as well as all applicable local laws and regulatory requirements, will be strictly followed to ensure the protection of study participants. The study protocol, informed consent form, and all other relevant study documents will be submitted by the site investigator to an institutional review board or independent ethics committee for review and approval prior to study initiation. Any protocol amendments will require prior approval from the institutional review board/independent ethics committee and relevant regulatory authorities, except when modifications are necessary to eliminate immediate hazards to participants. The current protocol version is version 1.0.

## Discussion

This study describes the design and implementation of a prospective, multicenter QIP aimed at standardizing hyperkalemia management in patients with non-dialysis CKD in China. The program integrates guideline-directed medical therapy with structured education for HCPs and patients, regular quality audits, and feedback mechanisms to improve serum potassium monitoring, promote sustained potassium control, and support optimization of RAASi therapy. By systematically applying evidence-based hyperkalemia management pathways over long-term follow-up, the study seeks to evaluate real-world care processes and clinically relevant outcomes related to potassium control, RAASi use, and cardiorenal health in a large, representative CKD population.

By implementing a standardized, guideline-based hyperkalemia management pathway within a QIP framework, this study has the potential to demonstrate that systematic potassium monitoring, proactive treatment of hyperkalemia, and coordinated education of HCPs and patients can meaningfully improve care processes in CKD. In particular, improved long-term potassium control may facilitate sustained optimization of RAASi therapy, thereby preserving its well-established cardiorenal protective effects. Beyond individual patient management, the multicenter design and integration of audit and feedback mechanisms may also lead to improvements at the institutional level, supporting more consistent adherence to guideline recommendations in routine clinical practice. These anticipated findings are consistent with emerging real-world evidence demonstrating that effective hyperkalemia treatment can enable continued or optimized RAASi use. In the OPTIMIZE I study, initiation of sodium zirconium cyclosilicate (SZC) in patients with hyperkalemia receiving RAASi was associated with RAASi optimization in nearly 80% of patients, with most maintaining or up-titrating RAASi doses and substantially higher long-term persistence compared with patients who did not optimize therapy [21]. Similarly, the GALVANIZE RAASi study showed that approximately two-thirds of patients achieved optimized RAASi dosing and more than one-quarter achieved maximized dosing within six months of SZC initiation, with consistent findings across subgroups with CKD, heart failure, and mineralocorticoid receptor antagonist use [22]. While these studies primarily focused on pharmacologic potassium-lowering interventions in claims-based populations, the present study extends this concept by evaluating a comprehensive, non-pharmacologic and pharmacologic, guideline-driven management strategy embedded within a QIP. As such, it may provide complementary evidence on how structured care pathways, beyond individual drug effects, can support RAASi optimization and improve long-term cardiorenal management in patients with CKD and hyperkalemia.

The potential value of the present study is further supported by evidence from prior QIPs across diverse clinical settings, which have consistently demonstrated that structured, audit-driven interventions can enhance guideline adherence and improve patient outcomes. For example, Luo et al. implemented a Plan-Do-Check-Act cycle-based QIP for cancer pain management and reported significant improvements in prescription compliance, patient satisfaction, and pain control [23]. Similarly, Abdulazim et al. showed that standardized, multidisciplinary management of delayed cerebral ischemia following aneurysmal subarachnoid hemorrhage was associated with reduced cerebral infarction rates and improved functional outcomes [24]. In China, the CARE4ALL study, the first nationwide asthma QIP, demonstrated that a structured QIP effectively narrowed the gap between guideline recommendations and routine practice, resulting in a 30.5% increase in asthma control rates and a reduction in exacerbations [25]. Collectively, these studies highlight the capacity of QIPs to translate evidence-based recommendations into sustained improvements in real-world care. By applying a similar structured approach to hyperkalemia management in CKD, the current study builds on this established body of evidence and addresses a critical unmet need in CKD care.

Several limitations of this study should be acknowledged. First, the single-arm design limits the ability to draw causal inferences regarding the effects of standardized hyperkalemia management on clinical outcomes, as there is no concurrent control group for direct comparison. Second, improvements observed during the study period may in part reflect increased clinical attention associated with participation in a QIP, consistent with a potential Hawthorne effect, whereby healthcare professionals and patients modify behavior due to awareness of being observed; this may have contributed to improvements in care processes and clinical outcomes independent of the standardized intervention itself. In addition, although the multicenter design enhances generalizability within China, the findings may not be fully applicable to other healthcare systems with different practice patterns or resource availability. Besides, objective quantification of dietary potassium intake, such as 24-hour urinary potassium excretion, was not routinely collected due to feasibility considerations in this large, multicenter quality improvement program; therefore, the potential impact of dietary potassium intake on serum potassium control and RAASi optimization could not be fully assessed. These limitations are partially mitigated by the use of predefined inclusion and exclusion criteria, standardized management pathways, objective outcome measures, and prolonged follow-up.

In conclusion, this study describes a nationwide, multicenter QIP aimed at standardizing hyperkalemia management in patients with CKD. By integrating guideline-directed care with structured education, regular audits, and feedback for HCPs and patients, the program seeks to improve potassium control and support sustained optimization of RAASi. The findings are expected to provide valuable real-world evidence on the feasibility and potential impact of systematic hyperkalemia management and may inform future strategies to improve cardiorenal outcomes in patients with CKD.

### Trial status

This is the final version of the protocol (version 6.0, dated December 2024). The first participant was enrolled on June 25, 2025, and the last participant is expected to be enrolled by January 24, 2026.

## Supplementary Information

Below is the link to the electronic supplementary material.

Supplementary Material 1

## Data Availability

The datasets generated or analyzed during this study will be available from the corresponding author upon reasonable request.

## Abbreviations

AEs: Adverse events
ADRs: Adverse drug reactions
CKD: Chronic kidney disease
FAS: Full analysis set
GCP: Good Clinical Practice
GDMT: Guideline determined medical therapy
eGFR: Estimated glomerular filtration rate
ESRD: End-stage renal disease
GFR: Glomerular filtration rate
ICF: Informed consent form
IRB: Institutional review board
IEC: Independent ethics committee
ICH: International Council for Harmonisation
MRA: Mineralocorticoid receptor antagonist
MPR: Medication possession ratio
MMRM: Mixed-effects model for repeated measures
RAASi: Renin–angiotensin–aldosterone system
RASi: Renin-angiotensin system inhibitors
SAS: Safety analysis set
SAEs: Serious adverse events
UACR: Urinary albumin/creatinine ratio
