# Supplementary material for: Study protocol for a prospective, multicenter, single-arm study investigating the impact of the implementation of standardized hyperkalemia management in chronic kidney disease patients
Source: BMC Nephrol. 2026 Apr 21;27:354. doi: 10.1186/s12882-026-04801-8 (PMC13237880; doi:10.1186/s12882-026-04801-8)
Supplement: Supplementary file 1 — Supplementary Material 1 [file 12882_2026_4801_MOESM1_ESM.docx]

**Supplementary materials**

**Patient selection criteria**

**Inclusion criteri**a

All patients must meet the following inclusion criteria to be eligible for participation:

1. Age ≥18 years at the time of informed consent signing.
2. Hyperkalemia (serum K^+^ > 5.0 mmol/L) within 48 h before enrolment.
3. Patients diagnosed with chronic kidney disease with estimated glomerular filtration rate (eGFR) >10 ml/min/1.73 m^2^ based on the Chronic Kidney Disease Epidemiology Collaboration (CKD-EPI) Creatinine Equation (2021).
4. Ability of giving signed informed consent which includes compliance with the requirements and restrictions listed in the ICF and in this protocol

**Exclusion criteria**

Patients will be excluded from the study if they meet any of the following criteria:

1. On dialysis.
2. Pseudo-hyperkalemia signs and symptoms, including hemolyzed blood specimen due to excessive fist clenching to make veins prominent, difficult or traumatic venipuncture, or a history of severe leucocytosis or thrombocytosis.
3. Acute kidney injury (AKI) or diabetic ketoacidosis (DKA).
4. Cardiac arrhythmias that require immediate treatment.
5. Scheduled renal transplant or a history of renal transplant.
6. Life expectancy < 48 weeks.
7. A history of malignancy except for:
   1. Malignancy treated with curative intent and no known active disease within 3 years before the enrolment and of low potential risk for recurrence.
   2. Adequately treated non-melanoma skin cancer or lentigo malignancy without evidence of disease.
   3. Adequately treated carcinoma in situ without evidence of disease.
8. Participation in other intervention clinical trials
9. Judgment by the health care professional (HCP) that the patient should not participate in the study if the patient is unlikely to comply with study procedures, restrictions, and requirements

**Table S1.** Contents of core slides for comprehensive and intensive training

| **Items** | **Highlights** |
| --- | --- |
| Disease education on hyperkalemia | Definition/Diagnosis/Initiation of Intervention:  • Hyperkalemia: sK^+^>5.0 mmol/L  HK management goals for CKD-ND patients:  • Reduce deaths; Ensure RAASi use to improve cardiorenal endpoint events (CKD patients who cut off RAASi therapy because of hyperkalemia have up to a 74% increased risk of ESKD progression) |
| Hyperkalemia follow-up and monitoring frequency | • Patients with CKD should have their potassium monitored at the initial diagnosis and at each subsequent review;  • In high-risk patients*, after the first potassium abnormality, the frequency of potassium monitoring should be increased (at least once a month) until the predisposing factor has been identified and addressed;  • Review sK^+^ to prevent hyperkalemia in CKD patients before and 1 to 2 weeks after starting RAASi administration or dose increases;  • (Hemodialysis and peritoneal dialysis patients should routinely have their sK^+^ checked every 1-3 months, and more frequent monitoring (at least monthly) is recommended if there is a history of hypokalemia or hyperkalemia). |
| Improvement for RAASi use in CKD patients via long-term management of hyperkalemia | • KDIGO CKD guidelines recommendations for RASi/MRA use;  • KDIGO CKD guidelines for response and management of the development of hyperkalemia after RASi/MRA: dose reduction and discontinuation of RAASi should be considered as a last resort, with first consideration given to correction of correctable factors and the use of potassium-lowering agents (e.g. novel K^+^ binders) |
| Treatment of hyperkalemia | • Dietary management: common hyperkalemic foods;  • Medication review: common drugs causing hyperkalemia and mechanisms  • Timing of initiation of non-pharmacological and pharmacological treatments, respectively (serum K^+^ levels)  • Characteristics and dosage of major potassium-lowering agents, etc. |

CKD-ND, non–dialysis chronic kidney disease; CKD, chronic kidney disease; RAASi, renin-angiotensin-aldosterone system inhibitor; ESKD, end-stage kidney disease; RASi, renin-angiotensin system inhibitor; MRA, mineralocorticoid receptor antagonist.

*High risk of hyperkalemia: CKD combined with DKD; CKD combined with HF, CKD with RAASi initiation or up titration

**Figure S1. Clinical pathway of non–dialysis chronic kidney disease patients with hyperkalemia.**

**
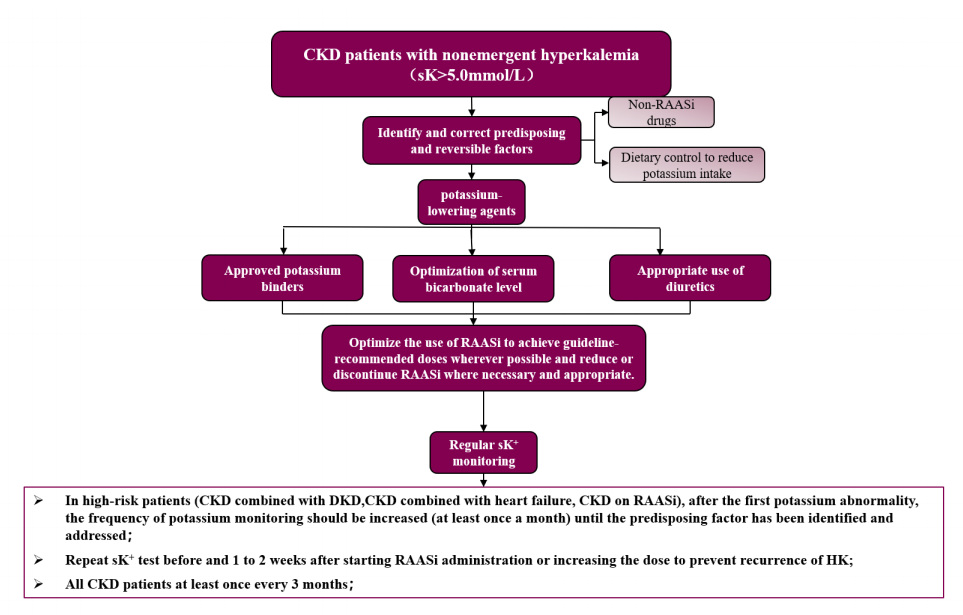
**

CKD-ND, non–dialysis chronic kidney disease; RAASi, renin-angiotensin-aldosterone system inhibitor; DKD, diabetic kidney disease; HK, hyperkalemia.
